# Crowdsourcing Medical Costs in Dermatology: Cross-sectional Study Analyzing Dermatologic GoFundMe Campaigns

**DOI:** 10.2196/34111

**Published:** 2022-04-22

**Authors:** Erica Mark, Mira Sridharan, Brian Florenzo, Olivia L Schenck, Mary-Margaret B Noland, John S Barbieri, Jules B Lipoff

**Affiliations:** 1 Department of Dermatology University of Virginia Charlottesville, VA United States; 2 Department of Dermatology University of Pennsylvania Philadelphia, PA United States; 3 Leonard Davis Institute of Health Economics Philadelphia, PA United States

**Keywords:** crowdfunding, crowdsourcing, fundraising, GoFundMe, social media, medical expenses, financial burden, health equity

## Abstract

**Background:**

Crowdfunding for medical costs is becoming increasingly popular. Few previous studies have described the fundraising characteristics and qualities associated with success.

**Objective:**

This study aimed to characterize and investigate the qualities associated with successful dermatological fundraisers.

**Methods:**

This cross-sectional study of dermatological GoFundMe campaigns collected data, including demographic variables, thematic variables using an inductive qualitative method, and quantitative information. Linear regression examined the qualities associated with success, which are defined based on funds raised when controlling for campaign goals. Logistic regression was used to examine qualities associated with extremely successful campaigns, defined as those raising >1.5 times the IQR. Statistical significance was set at *P*<.05.

**Results:**

A total of 2008 publicly available campaigns at the time of data collection were evaluated. Nonmodifiable factors associated with greater success included male gender, age 20-40 years, and White race. Modifiable factors associated with success included more updates posted to the campaign page, non–self-identity of the campaign creator, mention of a chronic condition, and smiling in campaign profile photographs.

**Conclusions:**

Understanding the modifiable factors of medical crowdfunding may inform future campaigns, and nonmodifiable factors may have policy implications for improving health care equity and financing. Crowdfunding for medical disease treatment may have potential implications for medical privacy and exacerbation of existing health care disparities. This study was limited to publicly available GoFundMe campaigns. Potential limitations for this study include intercoder variability, misclassification bias because of the data abstraction process, and prioritization of campaigns based on the proprietary GoFundMe algorithm.

## Introduction

### Background

Crowdsourcing medical expenses is an increasingly popular method of financing health care costs [1]. In particular, GoFundMe is the most popular crowdfunding website worldwide in terms of funds raised. As of 2021, one-third of the funds raised by GoFundMe (approximately US $650 million) are for medical campaigns [2]. In the United States, a staggering 62% of bankruptcies are related to medical costs [3]. The high financial burden of medical expenditures has contributed to the rise of popular crowdfunding sites such as GoFundMe [4]. Fundraising campaigns on GoFundMe are broadly advertised via social media outlets such as Facebook or Twitter, and potential donors are encouraged to share campaigns to increase visibility. By January 2020, 22% of American adults reported contributing to a GoFundMe campaign at least once, and 3% had created their own campaigns [5]. However, only approximately 10% of campaigns are successful in meeting their target goals [4]. With increased competition, campaigners are tasked with creating engaging and compelling appeals [4].

Limited research has considered the factors that influence the success of crowdfunding campaigns. Previous studies have suggested that demographic characteristics such as age and race, medical history, and proposed fund use are associated with fundraising outcomes, raising concerns about health care inequity and privacy [4-8]. Crowdfunding may be partly conceptualized as a marketing endeavor that requires creation of a campaign that will be seen as deserving to attract donations, especially if a medical condition is associated with any stigma. For instance, patients with lung cancer had more successful fundraising if they mentioned that they had never smoked, and patients with hepatitis C had more successful fundraising if they specified a source of infection that was ostensibly not intravenous drug use (blood transfusion, organ donation, and occupational exposures) [5,7]. Descriptive campaigns appear to raise more money, especially when patients provide a breakdown of specific medical and nonmedical expenditures; however, this may come at the expense of patient privacy [4-8]. We sought to analyze the specific themes most commonly associated with fundraising success when mentioned in campaign narratives. Previous studies have also suggested that racial minorities and older individuals are at a fundraising disadvantage [6,8]. Thus, in evaluating GoFundMe campaigns, we wished to evaluate any possible biases against marginalized groups, namely any gender-associated or race-associated biases.

### Objectives

Dermatological conditions may generally be viewed by the public with a low level of urgency [9]. However, 1 in 3 Americans may experience skin disease, and the direct costs associated with skin disease in 2013 were US $75 billion, with indirect costs (eg, loss of labor force) totaling US $11 billion [10,11]. We aim to characterize the fundraising campaigns on GoFundMe for dermatological conditions. Further, we sought to identify the qualitative themes and demographic variables associated with campaign success.

## Methods

### Ethics Considerations

This study was deemed exempt by the institutional review board of the University of Virginia.

### Data Collection

This study was deemed exempt by the institutional review board of the University of Virginia. We analyzed publicly available GoFundMe campaigns sorted by the platform algorithm from March 20, 2021, to May 31, 2021, until the completion of available qualifying campaigns using dermatology-specific search terms (dermatology, skin, cutaneous, dermatologist, rash, skin disease, skin infection, skin biopsy, finger and toenail infection, Mohs, scalp, alopecia, epidermal, dermal, birthmark, and skin cancer) chosen by author consensus. Exclusion criteria included campaigns outside the United States, recently activated GoFundMe campaigns (active <1 day), or if the primary reason for fundraising was not considered dermatologic. Demographic data pertaining to the beneficiary were either objectively mentioned or subjectively coded from the campaign text and images. Campaigns were classified under diagnostic categories based on the condition described and the intention for seeking treatment (eg, repair for cosmetic reasons vs functionality). Qualitative themes were coded using an inductive qualitative method until thematic saturation was reached, meaning that themes were continuously added as they appeared in the data until no novel themes emerged [12]. Each campaign was read completely by 2 independent coders and was associated with a maximum of 3 different themes.

### Statistical Analysis

The cleaned data were exported to RStudio (version 4.0.2). The frequencies of themes were calculated based on the percentage of times a theme was mentioned. Mann-Whitney *U* tests were performed for univariate analysis. Regression analyses were performed by comparing the number of shares and updates with the amount raised, controlling for race, age, gender, and campaign goal. A total of 2 separate models were used because of concerns regarding collinearity. Multivariable linear regression was performed to investigate the amount raised against the demographic and thematic variables. The Interquartile Method of Outlier Detection was applied to the amount raised and goal of the campaign. On the basis of this outlier detection method, campaigns raising >US $17,345 were excluded from the regression analysis. A binary logistic regression was run to compare demographic variables and themes in fundraisers that raised >US $17,345 with those that raised below this amount to investigate qualities associated with extreme success in fundraising. Extreme success was defined as an amount >1.5 times the IQR (>US $17,345). The significance threshold was set at *P*<.05.

## Results

### Demographic Variables and Campaign Summary

A total of 2008 fundraisers were analyzed. Most campaign recipients were White (1570/2008, 78.19%). There were more women (1109/2008, 55.23%) than men (896/2008, 44.62%). The campaigns raised a total of US $15,886,807 (mean US $7911.76, SD US $18,330.94, median US $3182) and had a total goal of US $45,860,361 (mean US $23,045.41, SD US $55,814.35, median US $10,000). A few campaigns met their goals at the time of the analysis (316/2008, 15.74%; Table 1).

**Table 1:** Demographic variables and campaign summary (N=2008).

Demographic variables			Values
**Gender, n (%)**			
	Female	1109 (55.23)	
	Male	896 (44.62)	
**Age (years), n (%)**			
	<10	343 (17.08)	
	11-20	163 (8.12)	
	21-40	911 (45.37)	
	41-60	466 (23.21)	
	≥61	120 (5.98)	
**Relationship status, n (%)**			
	Single	1273 (63.4)	
	In a relationship	735 (36.6)	
**Race, n (%)**			
	White	1570 (78.19)	
	African American	216 (10.76)	
	Asian	56 (2.79)	
	Hispanic	157 (7.82)	
	Other	9 (0.45)	
**Insurance status, n (%)**			
	Insured	813 (40.49)	
	Uninsured	220 (10.96)	
	Unclear	974 (48.51)	
**Top 5 most common themes for fundraising, n (%)**			
	Inadequacy of current insurance	1050 (52.29)	
	Medical condition limiting earning potential	601 (29.93)	
	Need to travel for care	448 (22.31)	
	Basic living expenses (utilities and food)	326 (16.23)	
	No insurance	213 (10.61)	
**Top 10 most common diagnoses, n (%)**			
	Melanoma	302 (15.04)	
	Nonmelanoma skin cancer	232 (11.55)	
	Alopecia	207 (10.31)	
	Road rash	117 (5.83)	
	Laceration	90 (4.48)	
	Burn	71 (3.54)	
	Systemic lupus erythematosus	64 (3.19)	
	Systemic scleroderma	61 (3.04)	
	Cellulitis	59 (2.94)	
	Lyme disease	56 (2.79)	
**Category of diagnosis, n (%)**			
	Malignant	615 (30.63)	
	Autoimmune	347 (17.28)	
	Traumatic	291 (14.49)	
	Infectious	206 (10.26)	
	Cosmetic	172 (8.57)	
	Inflammatory	168 (8.37)	
	Congenital	155 (7.72)	
	Outreach	54 (2.69)	
**Relationship to creator of campaign, n (%)**			
	Self	493 (24.55)	
	Partner	116 (5.78)	
	Family member	877 (43.68)	
	Friend	486 (24.2)	
	Other	36 (1.79)	
**Mention of religion, n (%)**			
	Yes	479 (23.9)	
	No	689 (34.31)	
**Amount raised (US $)**			
	Mean (SD)	7911.76 (18,330.94)	
	Median	3182.00	
**Goal of campaign (US $)**			
	Mean (SD)	23,045.41 (55,814.35)	
	Median	10,000.00	
**Number of updates**			
	Mean (SD)	4.24 (10.14)	
	Median	1	
**Number of donors**			
	Mean (SD)	89.96 (280.09)	
	Median	39	
**Number of shares**			
	Mean (SD)	529.34 (1035.47)	
	Median	232	

### Regression Analysis

The mean number of shares on social media was nearly 6 times the mean number of donations. Men had higher median shares (279, IQR 60.75-694.25) than women (201, IQR 18-492; *W*=424,586; *P*<.001) and more median donors (45, IQR 18-112) than women (35, IQR 12-69; *W*=414,304; *P*<.001). After adjusting for age, race, gender, and goal of the campaign, every additional share was associated with an additional US $6 raised for the recipient (*P*<.001) and each additional campaign profile update was associated with an additional US $262 raised (*P*<.001; Table 2).

With respect to demographic characteristics, Black recipients earned a mean of US $1146 less than White recipients (*P*<.001). Those in the age group of 41 to 60 years earned a mean of US $762 less than those in the 21 to 40 age group (*P*=.02). Men earned a mean of US $389 more than women did (*P*=.02). Those who mentioned the following themes received more donation money: medical conditions limiting earning potential (US $878; *P*<.001), need to travel for care (US $857; *P*<.001), complications from treatment (US $527; *P*=.04), funeral expenses (US $2013; *P*<.001), and having a chronic condition (US $622; *P*=.049). Smiling in profile photographs was associated with an earning mean of US $604 more than those without smiling (*P*=.01). Fundraisers created by friends earned a mean of US $1126 more (*P*<.001), and those created by someone other than a family member, friend, or partner earned a mean of US $1655 more than if created by the beneficiary themselves (*P*=.02; Table 3).

Age was a significant predictor of the likelihood of extreme success (defined as positive outlier campaigns raising >US $17,345) for those in the 21 to 40 age group, who raised more funds than those in the 61 to 80 age group (odds ratio [OR] 0.94, 95% CI 0.89-0.99). Men were more likely to experience extreme success than women (OR 1.04, 95% CI 1.01-1.06). Themes that were more frequently mentioned in the group with extreme success included the expressed loss of control (OR 1.150, 95% CI 1.012-1.306), chronic medical conditions (OR 1.060, 95% CI 1.012-1.110), need for medical equipment (OR 1.124, 95% CI 1.042-1.213), and rare medical conditions (OR 1.100, 95% 1.027-1.178). Themes that were less frequently mentioned in the group with extreme success included complicated comorbid conditions (OR 0.915, 95% CI 0.876-0.955). If the recipient was smiling in the profile photograph, the campaign was associated with an increased likelihood of extreme success (OR 1.032, 95% CI 1.002-1.061). If the relationship with the campaign creator was more peripheral or ill-defined, the campaign had a higher likelihood of extreme success (OR 1.170, 95% CI 1.061-1.292). An increase in the number of updates was seen in the group with extreme success (OR 1.006, 95% CI 1.005-1.007; Table 4).

Commentary associated with each theme is seen in Table 5.

**Table 2:** Linear regression of the amount raised association with number of shares and updates controlled for other variables^a^.

Dependent variable: amount raised		Shares				Updates		
		*β* (SE)	95% CI	*P* value	*β* (SE)		95% CI	*P* value
Number of shares or updates, respectively		5.729 (.2974)^b^	5.15 to 6.31	<.001	262.3 (31.71)^b^		200.08 to 324.45	<.001
Goal		.1743 (.0055)^b^	0.16 to 0.18	<.001	.1869 (.0058)^b^		0.18 to 0.20	<.001
**Age group (years; reference: 21-40 years)**								
	<10	−367.5 (854.4)	−2043.16 to 1308.21	.67	−185.3 (913.8)		−1977.38 to 1606.72	.84
	11-20	466.9 (1139)	−1757.57 to 2701.42	.68	−82.41 (122)		−2475.38 to 2310.55	.95
	41-60	−159.1 (767.1)	−1663.58 to 1345.31	.84	−1210 (819.9)		−2818.16 to 397.85	.14
	61-80	−1487 (1309)	−4053.90 to 1078.98	.26	−3501 (1394)^c^		−6235.36 to −766.78	.01
	>81	−946.5 (5963)	−12,640 to 10,747.78	.87	−3635 (6371)		−16,129.83 to 8859.52	.57
**Race (reference: White)**								
	African American	−3561 (988.4)^b^	−5499.47 to −1622.75	<.001	−3550 (1057)^b^		−5622.48 to −1477.06	<.001
	Asian	2547 (1808)	−998.40 to 6091.47	.16	2568 (1949)		−1253.48 to 6389.43	.19
	Hispanic	−1310 (1125)	−3516.84 to 896.70	.24	−461.5 (1203)		−2821.50 to 1898.57	.70
	Other	1274 (4442)	−7437.70 to 9985.73	.77	−13.21 (4747)		−9322.41 to 9295.99	.99
**Gender (reference: female)**								
	Male	927.3 (608.2)	−265.50 to 2120.19	.13	1841 (648.9)^d^		568.74 to 3113.79	.005

^a^Adjusted *R^2^* for shares=0.4687 and *R^2^* for updates=0.3865.

^b^*P*<.001.

^c^*P*<.05.

^d^*P*<.01.

**Table 3:** Multivariable linear regression of the amount raised by thematic and demographic variables of most campaigns^a,b^.

Dependent variable: amount raised		*β* (SE)	95% CI	*P* value
Goal		.210 (.016)^c^	0.19 to 0.23	<.001
**Age group (years; reference: 21-40)**				
	<10	393.5 (268.2)	−132.49 to 919.53	.14
	11-20	228.9 (322.0)	−401.66 to 860.43	.48
	41-60	−716.7 (216.2)^c^	−1185.74 to −337.69	<.001
	61-80	−417.5 (360.1)	−1123.84 to 288.80	.25
	>80	−2207 (1451)	−5053.80 to 640.06	.13
**Race (reference: White)**				
	African American	−1146 (270.0)^c^	−1675.96 to −616.76	<.001
	Asian	−690.6 (507.5)	−1686.09 to 304.93	.17
	Hispanic	−36.48 (305.7)	−636.07 to 563.11	.91
	Other	−872.2 (1323)	−3467.55 to 1723.15	.51
**Gender (reference: female)**				
	Male	389.2 (170.3)^d^	55.23 to 723.16	.02
**Fundraiser themes**				
	Loss of employment	567.6 (391.9)	−201.09 to 1336.37	.15
	Medical condition limiting earning potential	878.0 (186.1)^c^	512.93 to 1243.01	<.001
	Need to travel for care	857.3 (202.5)^c^	460.07 to 1254.61	<.001
	Complications from treatment	527.3 (255.1)^d^	26.94 to 1027.62	.04
	Funeral expenses	201.3 (519.1)^c^	995.08 to 3031.55	<.001
	Medical condition limiting activities	513.5 (281.4)	−38.40 to 1065.35	.07
	Chronic condition needing long-term treatment	621.5 (314.9)^d^	3.83 to 1239.23	.05
	Delayed medical attention	908.4 (491.7)	−56.14 to 1872.93	.06
	Money for childcare or family during treatment	−2316 (1318)	−4900.54 to 268.64	.08
**Fundraiser creator (reference: self)**				
	Family member	300.5 (230.6)	−151.91 to 752.90	.003
	Friend	1126 (240.1)^c^	655.51 to 1597.23	<.001
	Partner	−232.0 (407.8)	−1031.93 to 567.85	.57
	Other	1655 (682.2)^d^	316.64 to 2992.95	.02
**Miscellaneous**				
	Patient smiling	603.6 (182.6)^c^	245.46 to 961.74	<.001
	Patient single (reference: in relationship)	300.5 (230.6)^e^	−1017.49 to −211.27	.19
	Number of updates	87.99 (11.0)^c^	66.37 to 109.61	<.001

^a^Amounts raised >US $17,345 were excluded from analysis.

^b^Adjusted *R^2^*=0.316.

^c^*P*<.001.

^d^*P*<.05*.*

^e^*P*<.01*.*

**Table 4:** Binary logistic regression comparing campaigns with extreme success (>US $17,345 raised) with most campaigns by demographic and thematic variables^a^.

Dependent variable: amount raised >US $17,345 compared with below			*β* (SE)		Odds ratio (95% CI)		*P* value
Goal			.008 (.025)^b^		1.008 (0.961-1.058)		*<*.001
**Age group (reference: 21-40 years)**							
	<10	−.030 (.021)		0.970 (0.932-1.011)		.15	
	11-20	−.039 (.026)		0.962 (0.915-1.012)		.05	
	41-60	−.032 (.016)		0.968 (0.938-1.000)		.13	
	61-80	−.061 (.028)^c^		0.941 (0.891-0.993)		.03	
	>80	−.050 (.126)		0.951 (0.744-1.216)		.69	
**Race (reference: White)**							
	African American	.008 (.021)		1.008 (0.967-1.051)		.71	
	Asian	.075 (.038)^c^		1.078 (1.000-1.163)		.05	
	Hispanic	.010 (.024)		1.010 (0.964-1.058)		.68	
	Other	.126 (.094)		1.135 (0.945-1.363)		.18	
**Gender (reference: female)**							
	Male	.036 (.013)^d^		1.037 (1.010-1.064)		.006	
**Fundraiser themes**							
	Inadequate insurance or financial capacity	.022 (.013)		1.022 (0.996-1.049)		.09	
	Diagnostic difficulty	.045 (.024)		1.046 (0.999-1.095)		.06	
	Donation to charity or research	−.072 (.035)^c^		0.930 (0.868-0.997)		.04	
	Loss of family time	.049 (.033)		1.051 (0.985-1.120)		.13	
	Medical condition limiting activities	−.054 (.022)^c^		0.947 (0.908-0.989)		.01	
	Express loss of control	.140 (.065)^c^		1.150 (1.012-1.306)		.03	
	Chronic condition needing LT^e^ treatment	.059 (.024)^c^		1.060 (1.012-1.110)		.01	
	Need for medical equipment	.117 (.039)^d^		1.124 (1.042-1.213)		.003	
	Rare medical condition	.095 (.035)^d^		1.100 (1.027-1.178)		.007	
	At-home care expenses	−.059 (.032)		0.943 (0.886-1.003)		.06	
	Complicating comorbidities	−.089 (.022)^b^		0.915 (0.876-0.955)		*<*.001	
	Lacking self-confidence because of illness	−.043 (.027)		0.958 (0.909-1.010)		.11	
**Fundraiser creator (reference: self)**							
	Family member	.035 (.018)		1.035 (0.999-1.072)		.05	
	Friend	.031 (.019)		1.032 (0.994-1.071)		.1	
	Partner	.038 (.031)		1.039 (0.977-1.104)		.22	
	Other	.158 (.050)^d^		1.171 (1.062-1.292)		.002	
**Miscellaneous**							
	Patient smiling	.031 (.014)^c^		1.032 (1.002-1.061)		.03	
	Patient Single (reference: in relationship)	−.033 (.016)^c^		0.968 (0.938-0.999)		.04	
	Number of updates	.006 (.001)^b^		1.006 (1.005-1.007)		*<*.001	

^a^Nagelkerke *R^2^*=0.502.

^b^*P*<.001.

^c^*P*<.05.

^d^*P*<.01.

^e^LT: long-term.

**Table 5:** Representative quotes per thematic variable.

Variable	Participants^a^, n (%)	Quotes^b^
Inadequate insurance	1050 (22.9)	“The copay for each ER visit with my insurance is $450, not to mention the copays for all the follow-up visits. I have a $3000 deductible to meet before my insurance starts covering anything.”
Limited ability to work	601 (13.1)	“I also have had a difficult eczema-like rash for 2 weeks, which has prevented me from working my usual schedule.” (eczema) “Although he has insurance, his copays and travel expenses to visit specialists are quite significant. Because he works from home, this has limited his income.” (melanoma)
Travel	448 (9.8)	“The cost of specialists, labs, procedures, etc. really begin to add up. Not to mention the cost of transportation without a car.” “I can’t imagine what the medical bills will be, but the reality is that even now my mom is struggling to afford the daily parking fee to go see him.”
Money for basics (food, rent, and utilities)	326 (7.1)	“My primary concern is keeping the power and water on and food on the table.” (cellulitis) “They know that if the cancer does not devastate him, the inability to provide for his family might.”
No insurance	213 (4.6)	“I used to rely on Medicaid but now I don’t qualify since our income is too high. I am accumulating more debt on top of my old debt, so much so that I’m willing to tend to my own foot dressings and sutures.”
Complications from treatment	210 (4.6)	“They injected me with steroids, and I gained 100 lbs in less than one month. I developed huge stretch marks all over my stomach and legs. I can’t even bear to look at myself in the mirror.”
Limited activities	187 (4.1)	“I lost nearly all my friends since I was too sick to leave the house and they didn’t know how to deal with my chronic illness.”
Complicating comorbidities^c^	184 (4)	“As a diabetic patient, life has had its challenges. She deals with so much already. This is not what she needs right now.”
Chronic condition with need for long-term care	157 (3.4)	“Half of his life he has only known hospitals, needles and doctors and there is no end in sight, he needs help from people who want to help him.” (unspecified rash) The doctors say she could come home any day now but because she is going to require a lot of medical attention.” (systemic scleroderma)
Diagnostic difficulty	156 (3.4)	“Because typical mastocytosis is rare, not to mention the systemic form, doctors were skeptical, and thought she had an eating disorder. A lot of precious time was wasted.”
Self-esteem	133 (2.9)	“By reducing my scarring, I hope to bolster my self-esteem and move forward in both society and my career.”
Wig or hair prosthetic	100 (2.2)	“My wife is the most wonderful woman I’ve ever met, but I see the light in her eyes diminishing because of her hair loss. Wigs are very expensive.”
COVID-19	88 (1.9)	“Due to my condition, my fiancé had to take time off of work to care for our newborn. With this pandemic and a newborn baby, it is not easy to get child care at the moment. And because I am immunosuppressed, it adds new challenges for working outside of the home.”
At-home care expenses	87 (1.9)	“These funds will help pay for skilled home care as she adjusts to not being able to walk and learns how to regain her independence.” (systemic scleroderma)
Loss of employment	86 (1.9)	“Her employment has been terminated since she cannot provide them with a “reasonable” return date. Consequently, she will lose her medical coverage unless she pays more.”
Burden of previous debt	83 (1.8)	“I cannot afford to be afflicted with anything right now. I’m already behind on rent and bills.”
Loss of family time	77 (1.7)	“Not being together as a normal family has been tough on everybody to say the very least.”
Outreach	71 (1.6)	“Doctors are increasingly relying on private donations to continue their research and make progress in the field, and any dollar amount helps. The more people that see this, the closer we will be to finding answers!”
Rare medical conditions	69 (1.5)	“Since medical companies don’t make a profit off of rare diseases, they invest less in finding cures for these conditions. Insurance rarely covers cutting-edge treatments, and her doctors keep sending her for costly second opinions.”
Medical devices	59 (1.2)	“We want to provide him the independence he needs so he can live a normal life. Please help us get him a wheelchair he can operate himself (one-handed).” (epidermolysis bullosa)
Funeral expenses	55 (1.2)	“There still is a funeral to plan. Now we are asking for help for the funeral cost so we can put him to rest the way he would have wanted.” (epidermolysis bullosa)
Delay in medical attention	54 (1.2)	“She has struggled to get timely access to medications she needs to treat her disease. These delays—caused by a fundamentally broken health care and insurance system—have resulted in relapses of her disease and rejection by her body of the medications.” (psoriatic arthritis)
Trying to connect with people with similar diseases	22 (0.5)	“We are raising money so that she can attend an out-of-state conference about her rare condition in which many specialists will be presenting.” (epidermolysis bullosa)
Loss of control	19 (0.4)	“I am having trouble sleeping because I’m worried I’ll lose everything if my bills are not paid. My life revolves around cancer and worries like am I eating right, should I be exercising, how much sleep did I get, and what strange symptom do I have today? What does it mean? What is it from?”
End of life costs	15 (0.3)	“This fund has been created to support my father’s end of life costs. My siblings and I want to provide great hospice care and give him a proper send off.”
Preventative and alternative health	15 (0.3)	“To have a safe home they need an air filtration system, new windows. etc. to help decrease the number of allergens and bacteria within their home.”
Familial conflict because of disease	7 (0.2)	“I have been diagnosed with hypothyroidism and mast cell activation syndrome. Additionally, my spouse deserted me due to my chronic conditions knowing that as a stay-at-home mother I didn’t have an income of my own.”

^a^As campaigns endorsed multiple themes, and n reflects the total times a theme was endorsed, the total n does not equal the number of campaigns.

^b^Quotes have been paraphrased for anonymity and brevity.

^c^Complicating comorbidities refer to any expense incurred because of concurrent medical problems not associated with the primary disease stated in the fundraiser.

## Discussion

### Principal Findings

Our study identified factors associated with successful fundraising for dermatologic conditions on GoFundMe and specifically showed that thematic and demographic factors, including race and gender, have associations. Importantly, increasing the use of web-based crowdfunding introduces a new variable in the relationship between social media and medicine. The results of our study support the hypothesis that greater web-based social capital may be associated with successful fundraising. However, mobilizing these resources almost necessarily compromises patient privacy. Modifiable factors associated with success included a larger number of updates, non–self-identity of the campaign creator, mention of a chronic condition, and smiling in campaign profile photographs. Nonmodifiable factors associated with greater success included male gender, early to middle adulthood (age 21-40 years), and White race. Improved understanding of modifiable factors may guide future campaigns, and these identified nonmodifiable factors may have policy implications for improving health care equity and financing. Further, any reliance on crowdfunding to supplement insurance coverage highlights the potential shortcomings of the health care system and introduces questions regarding the balance between the risks and benefits for patients using social media to support their health care expenses. In particular, the identified nonmodifiable differences in crowdfunding may perpetuate the existing disparities in disadvantaged populations.

Social media literacy and robust web-based networks may increase the success of campaign fundraising. For every additional campaign profile update, fundraisers earned US $262 more per post, and for every additional share on social media, fundraisers earned US $6 more per post when controlling for race, age, gender, and goal of campaign. On an average, it took 6 shares to garner a single donation. Therefore, those with larger following on the web or followers with greater access to disposable capital may be at an advantage. Notably, higher income and educational levels have been associated with a larger number of donors and donation size in fundraisers for COVID-19 [6]. Together, these findings suggest that crowdfunded donations may be distributed inequitably, favoring the privileged [4,13]. Income and educational level were not available for analysis in our study and could provide further evidence to support this hypothesis. Access to technology, literacy, social capital, robust web-based networks, and self-marketing skills are factors that may contribute to a widening digital divide by enhancing opportunities to increase crowd appeal.

The need to mobilize these social networks and create an effective emotional appeal may undercut the right to medical privacy and patient autonomy. Campaigners noted detail information not only about their medical conditions but also personal expenses (Table 5). This information was provided voluntarily; however, pressure to increase appeal and legitimacy because of impending financial needs may undermine the right to medical privacy. The process of consent is also a concern when a campaigner is fundraising on behalf of a recipient and sharing second-hand personal information [14]. Interestingly, our study found that when the campaign creator was not the fundraising recipient, there was an association with increased success. Relationships that were more peripheral (friends) or ill-defined (others) had the greatest success. Potential donors may view fundraising by surrogates as credible evidence of increased disease severity, strong social ties that merit more donations, or an otherwise greater need for donation. Along the same line, other studies regarding GoFundMe success in patients with hepatitis C and lung cancer have shown that successful campaigns featured motifs emphasizing self-sufficiency, use of this platform as a last resort, framing the request for help as atypical, and highlighting that the individual was not at fault for their illness [4,7]. Campaigns that provided more information about etiology of disease and a breakdown of treatment costs were likely to receive higher donations [4]. GoFundMe encourages the release of this information through their “Top Tips” page, which includes recommendations for frequent updates, inclusion of ≥5 images, and divulsion of details regarding the recipient’s personal life and medical treatment [2]. Other studies have similarly noted the trend of including extensive personal information, with some advocating for GoFundMe to change their recommendations; institute a consent process for fundraising on behalf of others; and obtain a release for personal information or restrict information posted without consent [6,14].

Medical fundraising campaigns may affect the relationship between physicians and patients on social media. For instance, campaigns may mention physician names and private medical details to increase campaign legitimacy. Jia et al [15] found that if the physician’s name was mentioned in melanoma campaigns, the amount raised was doubled. Other studies have noted concerns over the use of GoFundMe without physician supervision as it may promote unfounded medical treatments [16-18]. Currently, it is not common practice for patients to consult physicians about information shared via social media. If physicians see their obligation to their patients as maximizing patient benefits and minimizing harm, this implies that physicians may choose to expand their roles as patient consultants in web-based and social media venues. However, it is worth noting that this raises further questions regarding physician privacy and traditional professional boundaries.

Disclosure of a chronic medical condition was another modifiable variable associated with increased success in both regressions. Previous reports have recognized that individuals with chronic conditions often have unmet needs within the American health care system [19]. Furthermore, chronic rather than acute conditions are hypothesized to more strongly invoke the sick role and increase donor sympathy [19]. Some believe that this phenomenon occurs because of reinforcement of the concept that the resolution of chronic disease is unexpected and thus may be costlier [20]. Consistent with other studies on GoFundMe donations, the success of campaigns citing this theme may be related to creating an image of deservingness and emphasizing the lack of culpability in their disease processes or financial situations [4,7]. This knowledge could potentially be applied to educate patients seeking to maximize their returns from GoFundMe fundraising. Similar to many profit-based endeavors, improving social media skills and expertise could assist patients in increasing fundraising success through comprehension of which qualities to emphasize and which to avoid.

Along these lines, smiling in campaign profile photographs was also associated with increased success, suggesting the benefits of strategized visual campaign curation. Other studies have theorized that this effect may be because of observers mimicking the emotions depicted in images, thus motivating donations to maintain these sentiments [21,22]. Smiling may also influence the perceived attractiveness of a recipient. Previous research suggests that the perceived attractiveness of female recipients may lead to larger donations [23]. These observations, in conjunction with the fact that this study found men to be more likely to achieve campaign success, may have ethical implications regarding distributive justice and evoke concerns about unconscious biases in crowdfunding. Canadian researchers have suggested that, paradoxically, although campaigns are typically created in response to known gaps in the social system, the resulting campaign outcomes reinforce rather than rectify established socioeconomic disparities [6,8]. If health care financing shifts from an institutionalized to an individual system, resources may be distributed not based on need but rather based on social worthiness or appeal.

In both regressions, the goals of campaigns were related to increases in the amounts raised. There are limitations to interpreting this relationship, given several confounding factors. Those with higher goals are less likely to meet their fundraising ceilings. In theory, having a high unmet goal could potentially encourage additional donations until an inflection point is reached, and these exceptionally high goals may seem futile and unobtainable for donors. In addition, higher goals may reflect disease states of greater severity and need. Conditions that are more severe may inherently have a greater crowd appeal and contribute to the higher amount raised.

Regarding nonmodifiable variables, our study suggests that demographic differences, including race, age, and gender, affect fundraising. Black, female, and older patients were all less successful in their fundraising campaigns. Kenworthy et al [4] also found that, although women were less likely to be as successful as men in fundraising, women created most fundraisers. In this study, men also had more shares and donations than women. Notably, trends in fundraising success within the limited landscape of GoFundMe may not mimic trends in earning potential and health care burden seen in society at large. Previous studies have found that compared with their White counterparts, people of color are more likely to be both underinsured and experience adverse health outcomes [24]. In addition, according to the Pew Research Center, the salary of American women in 2020 was 84% of the salary earned by men [25]. Older individuals have more limited income opportunities and are also more likely to experience medical conditions, particularly skin cancers [26]. These differences may be exacerbated by the increased burden that traditionally marginalized groups (ie, older patients, racial minorities, and individuals of lower socioeconomic status) have accessing web-based resources, thereby leading to smaller web-based social networks and influence. In interpreting these findings, it is imperative to question the role that donor bias may play in fundraising success. Unconscious bias regarding darker skin tones has been associated with lower fundraising amounts, even when controlling for donor education, race, gender, political ideology, and past giving behavior [27]. Although gender and age biases against women and older individuals in nonmedical fundraising have been documented, controlled experiments to evaluate unconscious biases in health care crowdfunding are needed [4]. Given that these specific populations, on average, earned less money fundraising, these observed trends suggest that patients with the greatest need for financial assistance may be particularly disadvantaged.

Although increased reliance on crowdfunding for medical expenses could be criticized as a natural consequence of an imperfect health care system failing to meet the needs of a large segment of the population, crowdfunding may currently serve a purpose as a social safety net for those facing financial hardship. However, to ensure parity and that any social safety net provides coverage for those who need it the most, future work should continue to explore the amount of invested labor and derived benefits for all demographic groups.

### Limitations

This study was conducted using data from GoFundMe. Future studies are needed to examine whether these findings can be generalized to other crowdfunding platforms. There is a possibility of misclassification bias as the authenticity of each campaign could not be verified. In addition, age could only be evaluated as a categorical variable as many patients referenced their decade of life but not specific ages. There is also the possibility of misclassification because of the data abstraction process; however, each post was reviewed by 2, reviewers and entries were discussed as a team to minimize the potential introduction of bias. Furthermore, GoFundMe does not release the proprietary algorithm that guides search tools; as only the first 960 campaigns per search term are displayed, it is possible that some campaigns could not be assessed depending on how GoFundMe’s search algorithm prioritizes different content. Finally, it is worth noting that our study coincided with the COVID-19 pandemic. Although mentions of COVID-19 were not significantly associated with campaign success, future studies should seek to explore the crowdfunding frequency and success of campaigns coinciding with the pandemic.

### Conclusions

The results of this cross-sectional study suggest that dermatologic crowdfunding success is associated with modifiable and nonmodifiable variables such as race, gender, and age. Improved understanding of modifiable factors may guide future campaigns, and identified nonmodifiable factors may have policy implications for improving health care equity and financing. GoFundMe may have the potential to exacerbate and introduce health care inequalities skewed along the lines of these factors and web-based social capital. However, identifying the factors associated with successful fundraising and social media education may assist patients in self-advocacy. Future research should further investigate the impact of GoFundMe campaigns in the medical field.

## Abbreviations

OR: odds ratio
